# Doctrines and Practice of Hippocrates in Surgery and Physic; with Occasional Remarks

**Published:** 1785

**Authors:** 


V. *Doctrines and Practice of Hippocrates in Surgery and Phyſic; with occaſional remarks. By* Francis Riollay, *M. B.* 8vo. Cadell, *London*, 1783. 273 pages.

THE principal object of M. Le Clerc, as a medical hiſtorian, was to repreſent, in a general manner, the ſtate of Phyſic in the time of Hipprocrates. The profeſſed deſign of the work now before us, is to determine how far a ſtudy of the writings of Hippocrates is uſeful at preſent; and at the ſame time to leſſen the labours of medical readers, by freeing them, in a great meaſure, from the unneceſſary minuteneſs, frequent obſcurities, contradictory paſſages, and confuſed manner, with which their worth is mingled and disfigured.

The

The work is divided into two parts, each of which is fubdivided into chapters. In the firft part, Dr. Riollay reviews the furgical, and in the fecond, the medical writings of Hippocrates. He has defignedly (and for very good reafons, which are given in the work) fuppreffed his fyftem of generation, and four or five little tracts concerning the nature of children, dentition, anatomy, &c. Of all the reft he has given fome extracts, more or lefs confiderable, according to his notions of their intrinfic value, or of their confiftency with his plan.

In his remarks on the critical days, he offers many judicious arguments to prove that the generally-received doctrine on this fubject has no foundation in nature.

As a fpecimen of the defcriptive manner of Hippocrates, Dr. Riollay has chofen to give a tranflation of the firft book of epidemics, rather than extracts of all the feven books. Thefe, though the moft celebrated of all Hippocrates' writings, do not feem calculated, in our author's opinion, to improve either our difcernment in diftinguifhing, or our fkill in the treatment of difeafes. Dr. Riollay very properly allows, that a good defcription of difeafes, which, either from a common fource of contagion, or a peculiar ftate of the air, fpread themfelves extenfively without any material difference in the fymptoms, may be truly valuable; but that to minute down every variation of weather glaffes, to keep a daily lift of every patient's diftemper, and communicate all this under the name of epidemics, is not likely to prove of greater advantage to phyfic than the annual publication of the bills of mortality.

Of the aphorifms of Hippocrates, Dr. Riollay remarks, that, generally fpeaking, they fhew a great acquaintance with difeafes, and a wonderful habit of obfervation; but, at the fame time, an unphilofophical haftinefs to convert particular inftances into fixed laws of nature. "Some," he obferves, "are fo remarkably "juft, that they feem to have been the refult "of very long experience; whilft others are "as evidently falfe, and cannot have been "tranfmitted to us by the fame pen."— "Many," adds our author, "are uncertain; "fome (and he particularly inftances the "fourth aphorifm of the fifth fection) are "abfurd; and not a few, unintelligible."

From the whole of this review, Dr. Riollay ventures to conclude, that Hippocrates contributed but little towards the inveftigation of difeafes, difeafes, or the introduction of effential remedies; and he thinks it probable, that our ignorance of the ftate of phyfic before his time, and the difcovery, that his writings were the fountain of the Arabian doctrines, are the two grand caufes of his extraordinary reputation among us. At the fame time, however, he candidly acknowledges, that the works of this celebrated ancient, amidft a great deal of uninftructive, unneceffary matter, afford evident marks of extenfive knowledge, ftrong underftanding, genius for obfervation, an enlarged manner of thinking, and profeffional candour.

